# Breast Density Assessment Using a 3T MRI System: Comparison among Different Sequences

**DOI:** 10.1371/journal.pone.0099027

**Published:** 2014-06-03

**Authors:** Alberto Tagliafico, Bianca Bignotti, Giulio Tagliafico, Davide Astengo, Lucia Martino, Sonia Airaldi, Alessio Signori, Maria Pia Sormani, Nehmat Houssami, Massimo Calabrese

**Affiliations:** 1 Institute of Anatomy, Department of Experimental Medicine, University of Genova, Genova, Italy; 2 Radiology Department, University of Genova, Genova, Italy; 3 CNR-IMATI, Consiglio Nazionale delle Ricerche, Istituto di Matematica Applicata e Tecnologie Informatiche, Genova, Italy; 4 Institute of Statistics, Department of Health Sciences, University of Genova, Genova, Italy; 5 Screening and Test Evaluation Program (STEP), School of Public Health, Sydney Medical School, University of Sydney, Sydney, Australia; 6 Department of Diagnostic Senology, Ist Istituto Nazionale per la Ricerca sul Cancro, IRCCS Azienda Ospedaliera Universitaria San Martino, Genova, Italy; University Medical Center (UMC) Utrecht, Netherlands

## Abstract

**Purpose:**

To compare MRI sequences for breast density measurements on a 3T MRI system using IDEAL (Iterative Decomposition of water and fat with Echo Asymmetry and Least squares estimation) as possible physiology-like reference.

**Materials and Methods:**

MRI examination was performed in 48 consecutive patients (mean age 41, years; range, 35–67 years) on a 3.0T scanner and 46 were included. All (fertile) women, were examined between days 5 and 15 of their menstrual cycle. MRI protocol included: T1-turbo spin-echo (T1-tSE), T2-turbo spin-echo (T2-tSE), VIBRANT (Volume Imaging for Breast Assessment) before and after injection of contrast media and IDEAL. Breast density was calculated with semi-automated software. Statistical analysis was performed with non-parametric tests.

**Results:**

Mean percentage of breast density calculated in each sequence was: T1-tSE  = 56%; T2-tSE  = 52%; IDEAL FatOnly  = 55%; IDEAL WaterOnly  = 53%, VIBRANT  = 55%. Significant differences were observed between T2-tSE and both T1-tSE (p<0.001), VIBRANT sequences (p = 0.009), T1-tSE and both IDEAL WaterOnly (p = 0.007) and IDEAL FatOnly (p = 0.047). Breast density percentage showed a positive linear correlation among different sequences: r≥0.93.

**Conclusions:**

Differences exist between MRI sequences used to assess breast density percentage. T1-weighted sequences values were similar to IDEAL sequences.

## Introduction

Breast density is a factor that should be taken into consideration when evaluating breast cancer risk. Breast density is an independent risk factor for cancer [1]: it has been demonstrated that women with dense breasts have a 4–6 times higher risk than women with fatty breasts [2]–[4]. A quantitative evaluation of breast glandular tissue is also very important for epidemiological studies [5], [6]. Cancer risk predictive models, such as the Gail model, improve their predictive accuracy when breast density is incorporated [7], [8]. To date, there are different density classification methods like Tabar's classification [9], [10], Wolfe's parenchymal patterns [11] and several quantitative evaluations of percentage mammographic density using automated or semi-automated computer-aided techniques [12]–[16]. However, the practical and logistical difficulties in correlating histopathological samples of the breast with radiological images prevent the definition of a gold standard for breast density evaluation. Therefore, a standard classification method to evaluate breast density is a critical challenge, in particular for Magnetic Resonance Imaging (MRI)[17], [18]. MRI has been used for breast density assessment [19] and correlation has been demonstrated between breast density percentage on MRI and mammography [17], [20]–[24]. Other techniques, like mammography or tomosynthesis, could be adjusted on the basis of MRI for the evaluation of breast density percentage [25]. MRI assessment of breast density has been estimated using different sequences. Since 2007, sequences that can clearly separate the fatty non-glandular tissue from the true glandular tissue and its water content have been developed and made available for clinical purposes [26]. These sequences are called IDEAL (Iterative Decomposition of water and fat with Echo Asymmetry and Least squares estimation) and are believed to assess directly the biochemical features and composition of breast tissue similarly to physiology although they are still a surrogate of the real histological reference standard [26]. Other MRI sequences employed in the past evaluated breast density with proton density principles, as for mammography and digital breast tomosynthesis [25], [27]. To our knowledge, there are no data in the literature comparing standard MRI sequences and IDEAL sequences for breast density percentage evaluation on a 3T MRI system. Therefore, the purpose of this study was to compare breast density percentage assessment with different MRI sequences considering IDEAL sequences (Iterative Decomposition of water and fat with Echo Asymmetry and Least squares estimation) as a possible physiology-like reference.

## Materials and Methods

### Ethics Statement

The study was approved by the Local Ethic Committee (National Institute for Cancer Research) and written informed consent was obtained from all participating women.

### Patients

48 consecutive patients (mean age 41, years; range, 35–67 years; mean weight (kg) 56±4; mean BMI 22±3) underwent MRI examination, from March 2010 to October 2012. All women, if fertile, were examined between days 5 and 15 of their menstrual cycle. MRI examinations were performed after clinical and radiological examination, following EUSOMA (European Society of Breast Cancer Specialists) Guidelines [28] which were considered the inclusion criteria. Exclusion criteria were: breast prosthesis, bilateral breast pathology, claustrophobia, inability to tolerate MRI and lack of written informed consent.

### MRI

MRI examinations were performed on a GE Signa HDx 3.0T scanner (General Electric Medical Systems, Milwaukee, WI, USA) with a dedicated eight-channel bilateral breast coil: the patient was placed in a prone position, without any compression of breasts. MRI scan protocol included the following standard sequences: T1-tSE, T2-tSE, pre- and post-contrast agent (Gadobenate dimeglumine, MultiHance 0,5 M, Bracco, Italy) VIBRANT (Volume Imaging for Breast Assessment) with the adjunct of IDEAL (Iterative Decomposition of water and fat with Echo Asymmetry and Least squares estimation) sequences [25], [29]. IDEAL sequences are based on Dixon's method for fat suppression with the “in- and out-of-phase” technique: two images are acquired with different echo times (TE). Differences in chemical shift between fat and water should permit a clear separation of these tissues in perfect conditions [30]. This method was progressively modified and improved with the reduction of B0 field inhomogeneities [26], [31], [32]. IDEAL sequences were included in this protocol to distinguish fat tissue from water (FatOnly sequences and WaterOnly sequences). WaterOnly sequences were considered to represent glandular tissue since fat is a water poor tissue. These sequences correlate well with physiology, but they are considered a surrogate for the real histological reference standard [26]. Main sequences parameters are reported in Table 1.

**Table 1:** Sequence parameter settings.

Sequence	TR (ms)	TE (ms)	Flip angle	Matrix	Slice thickness (mm)	Spacing (mm)	Approximate Scan time (s)
T2	5200	103	90°	350×350	4.0	3.0	200
T1	600	9	90°	350×350	4.0	3.0	300
VIBRANT	6.2	3	10°	350×350	1.2	1.2	90
IDEAL	4380	130	90°	360×360	1.2	3.0	3600

IDEAL :Iterative Decomposition of water and fat with Echo Asymmetry and Least squares estimation. VIBRANT: Volume Imaging for Breast Assessment.

s = seconds, mm = millimeters, ms = milliseconds

### Breast density analysis with semi-automated software

**Figure 1:**
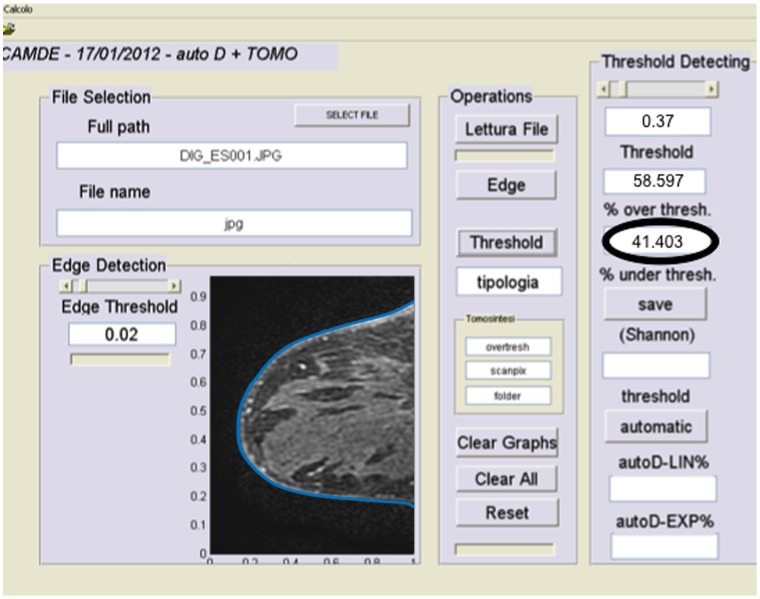
Quantitative assessment of breast density using the thresholding method. In this figure an example of the graphical computer interface is demonstrated. On the left, the edges of the breast are identified (edges are artificially thickened for visual purposes), then the radiologist adjusts the density threshold (semi-automated method) on the right, and finally the percentage of breast density is shown (*black circle*). In this example, the breast result was 41% dense.

Breast density analysis was performed only on the unaffected breasts. The data set comprised MRI images obtained in 48 patients. Two patients were completely excluded for bilateral pathological findings. In the other 46 patients, the breast affected by cancer, fibroadenomas and cysts was excluded to avoid interference with the breast density analysis. There were 12 patients screened for BRCA-1 mutations and had negative results after MRI, 25 patients with unilateral breast cancer (22 invasive ductal carcinomas and 3 invasive lobular carcinomas), and 9 with unilateral pre-malignant lesions. Every MRI sequence was evaluated before the administration of contrast agent to avoid possible influences of Gadolinium on breast density assessment. Breast density was calculated for each sequence using semi-automated software (MedDensity^©^). This software is a home-grown and previously validated for mammography, tomosynthesis and MRI [15], [16], [25]. All slices of every MRI sequence were included. We used the semi-automated version of the software (Fig. 1) because it permits proper tuning by the reader to guarantee more precise results. The software was adjusted to reduce user-related variability in assessing breast density with different sequences. The evaluations were performed by two radiologists independently (M.C. and A.T.). The two radiologists had 22 and 7 years respectively of experience in breast imaging and with more than 80 breast MRI reported every year. After the first evaluation, measurements were repeated after 2 months to assess intra- and inter-observer variability.

### Statistical analysis

Statistical analysis was performed with commercially available softwares IBM SPSS Statistics v.19 (International Business Machines Corp., New York, NY, US) and R (http://www.r-project.org/). After preliminary data checks, by mean of histograms, skewness and kurtosis index, the normality of distribution of each sequence, the non-parametric Friedman test was used to compare the five MRI sequences. If an overall significant result was detected, paired comparisons between the sequences were performed using the non-parametric Mann-Whitney test for paired samples. A p-value <0.05 was considered statistically significant. For multiple comparisons p-value was adjusted using the Holm correction criteria. Intra- and inter-observer variability were calculated using K statistics. Agreement was defined on the basis of Fleiss classification as follows: <0.40, poor; 0.40–0.59, moderate; 0.60–0.75, good; >0.75, excellent [28], [33], [34]. In addition, we considered in the analysis the effects of user interpretation and manual selection of threshold as confounding factors and adjusted the software interface and the statistical significance accordingly: p values <0.01 were considered statistically significant for that specific purpose.

## Results

Forty-six women were included in the study. Mean percentage of breast density calculated in each sequence was: T1-tSE  = 56%; T2-tSE  = 52%; IDEAL FatOnly  = 55%; IDEAL WaterOnly  = 53%, VIBRANT  = 55% (Table 2). Breast density percentage showed positive linear correlation among different sequences: *r* = 0.95 between T1-tSE and T2-tSE; *r* = 0.93 between T1-tSE and FatOnly; *r* = 0.93 between T2-tSE and FatOnly (we reported the maximum and minimum values of *r*).

**Table 2:** Mean, median, maximum and minimum values of breast density percentage evaluated on every MRI sequence with the semi-automated software.

Sequence	Mean	Median	Maximum	Minimum	Standard Deviation
T2	52	58	83	6	22
T1	56	61	95	7	23
VIBRANT	55	56	89	8	23.2
WaterOnly	53	534	95	3	23.1
FatOnly	55	54	92	3	23

Results include both readings from both radiologists. IDEAL sequences (Iterative Decomposition of water and fat with Echo Asymmetry and Least squares estimation) are reported are WaterOnly and FatOnly. VIBRANT: Volume Imaging for Breast Assessment.

Comparing the five MRI sequences, statistically significant differences were observed between T2-tSE and both T1-tSE (p<0.001) and VIBRANT sequences (p = 0.009 using Student's T-test; p = 0.002 using non-parametric test). Complete results are shown in Table 3.

**Table 3:** Test for paired samples to evaluate mean value differences between results of breast density percentage obtained on each MRI sequence.

Sequence Comparison	Differences in pairs				P-value
	Mean	SEM	95% CI		
			Inferior	Superior	
T2 - T1	−4.25	.98	−6.22	−2.27	.000
T2 - VIBRANT	−2.85	1.04	−4.94	−.75	.009
T2 - WaterOnly	−1.46	1.18	−3.84	.92	.225
T2 - FatOnly	−2.00	1.52	−5.06	1.06	.195
T1 - VIBRANT	1.39	1.24	−1.10	3.90	.268
T1 - WaterOnly	2.78	.90	.97	4.60	.003
T1 - FatOnly	2.31	1.19	−.08	4.71	.058
VIBRANT - WaterOnly	1.39	1.05	−.73	3.52	.195
VIBRANT - FatOnly	.87	1.33	−1.82	3.56	.518
WaterOnly - FatOnly	−.55	.60	−1.76	.66	.365

95% C.I. and P-value have also been calculated. IDEAL sequences (Iterative Decomposition of water and fat with Echo Asymmetry and Least squares estimation) are reported as WaterOnly and FatOnly. VIBRANT: Volume Imaging for Breast Assessment.

SEM =  Mean Standard Error, 95% CI =  Confidence Interval for the difference.

Further statistically significant differences were observed between T1-tSE and both WaterOnly (p = 0.007) and FatOnly (p = 0.047). Complete results are shown in Table 4.

**Table 4:** Wilcoxon Test comparing breast density percentages evaluated on different MRI sequences (^a.^ based on negative ranks; ^b.^ based on positive ranks).

	T1 - T2	VIBRANT - T2	WaterOnly - T2	FatOnly - T2	VIBRANT - T1	WaterOnly - T1	FatOnly - T1	WaterOnly - VIBRANT	FatOnly - VIBRANT	FatOnly -WaterOnly
Z	−3.856a	−3.063a	−1.357a	−1.195a	−1.114b	−2.687b	−1.988b	−1.571b	−.525b	−1.086a
P-value	.010	.002	.175	.232	.018	.007	.047	.116	.600	.278

VIBRANT: Volume Imaging for Breast Assessment.

Intra-observer and inter-observer agreement of the two radiologists in the evaluation of breast density were considered to be very good (reader 1: k = 0.93; reader 2: k = 0.95; reader 1 vs reader 2: k = 0.95).

## Discussion

Given the increasing importance of breast density percentage assessment in both the clinical and research fields, the development of a reliable and reproducible evaluation method would be useful [15]. Breast density percentage assessment has a very relevant role in life-time cancer risk calculation [1−4], therefore its inclusion in predictive models may improve their accuracy [7], [8].

To date, many methods for breast density assessment are based on the evaluation of analogic or digital mammography, but none of them are calibrated with a reference standard similar to histology. Using a method that is able to assess breast density percentage close to the real histological composition of the breast may be important to achieve a breast density percentage assessment similar to the real anatomy of breast tissue. Indeed, histology would give a true representation of the breast density, but histological specimens are generally not available for practical reasons. Recently, MRI proved to be a potentially reliable technique for this purpose [27]. Three-dimensional MRI imaging of the breasts may be better than two-dimensional techniques similarly to other 3D imaging techniques that may be superior to 2D evaluations [35]. In the literature, other authors evaluated breast density with MRI. Most of them used standard T1-weighted sequences, yet it has not been proven whether or not this evaluation resembled the real composition of breast tissue. In particular, various researchers have used the following sequences for breast density assessment: Khazen et al. used pre-contrast T1-weighted [17], Lee et al. used T1-weighted spoiled gradient echo fast low-angle shot [22], Klifa et al. used 3D fat suppressed spoiled gradient echo pulse [19], [36], Wei et al. used coronal 3D spoiled gradient recalled echo pre-contrast T1-weighted [23], Thompson et al. used pre-contrast T1-weighted [37], Eng-Wong et al. used T1-weighted spoiled gradient-echo with fat suppression [38], and Wang et al. used a T1-weighted non-contrast fat-saturated images [39]. A common limitation of all these studies was that none of them used a sequence like the IDEAL that may be more likely to resemble the true breast tissue composition. These IDEAL sequences may be used as a physiology-like reference. A comparison between results obtained from MRI and mammographic examination demonstrated that MRI and mammography had good correlation only in low-density breasts (BI-RADS 1 and 2). In women with a greater breast density the correlation between MRI and mammography was low [36]. Evaluation of breast density on mammograms has limitations and technical problems. For this reason a three-dimensional imaging technique may be more accurate [40]. In the present study we added IDEAL sequences to study breast tissues from a biochemical perspective and not only according to the proton density typical of T1weight [25], [27]. This sequence may represent a reference in the assessment of breast density percentage because it is able to separate FAT and WATER. The main disadvantage of IDEAL sequence is longer scan time compared to other fat-suppression techniques [26]. Comparing IDEAL sequences with other MRI sequences in our study protocol (T1- and T2-weighted and VIBRANT), we found that there were differences in breast density percentage assessment. Breast density percentage evaluated on T1-tSE and IDEAL WaterOnly (our reference standard for breast density) were similar. On the contrary, percentages evaluated on T2-tSE and VIBRANT sequences were different from IDEAL WaterOnly. T1-tSE showed similarity in the assessment of breast density percentage to IDEAL WaterOnly. From these data we suggest that T1-weighted sequences produced images that are similar to the composition of breast tissues. The reliability of T1-weighted sequences in the assessment of breast density is enhanced by our data. A T1-weighted sequence is often included in the standard breast MRI protocol, while IDEAL sequences are not so widely available and increase the duration of the examination. For this reason we suggest that T1-weighted sequences are sufficient to assess breast density on MRI.

This study has several limitations. All examinations were performed on a GE Signa HDx 3.0T scanner but we suppose that for breast density assessment similar results are likely to be obtained on a lower magnetic field scanner. This assumption is supported by the fact that T1-weighted sequences, for breast density assessment, are relatively stable at 1.5T and 3.0T. We acknowledge that further research could be performed to investigate this hypothesis. IDEAL sequences work better on a 3.0T than on a 1.5T magnetic field because the chemical shift between water and fat is increased and consecutive echo groups have smaller spacing between them [26]. Image quality on 3.0T with a well-defined contrast among different tissues is improved. In our study we used semi-automated software because superiority control and regulations by reader was considered essential due to artifacts related to B0 and B1 inhomogeneity [41]. A fully-automated software could have reduced time, costs and might have guaranteed more reproducible results, however an accurate control by the reader was sufficient to achieve similar results between different readings. Intra- and inter-observer agreement was very good and it is unlikely that the human reader may have influenced the results significantly. The software which was used was straightforward and easy to use and very little training was needed. No statistically significant differences between the first and the second reading performed after two months was found. For this reason radiologists' expertise in breast density assessment had little impact. Concerning inclusion criteria we used EUSOMA Guidelines [28], therefore our patients represent a selected population of women for whom MRI was clinically suggested based on these guidelines. Institutions with different inclusion criteria may have a different patient population. This is the first study of this kind and our data were statistically significant even with a relatively small number of patients. Another limitation could be the artifacts related to B1 inhomogeneity which should be considered when using a 3T MRI system. All the sequences were acquired consecutively on the same patient to reduce the measurement error due to B1 artifacts. B1 field homogeneity does not depend on body type and it is significantly improved by performing local RF shimming. Our MR system performs routinely local RF shimming for breast MRI. In the future, the value of a subject specific dual-transmit approach for improving B1 field homogeneity should be assessed for breast MRI. It has been demonstrated that with this approach, B1 field homogeneity is significantly improved by performing local radiofrequency shimming with 2 independent radiofrequency-transmit channels [42]. In our study there was not a shimming with a dual-transmit system to improved local radiofrequency homogeneity, but only a standard shimming. Further research is needed to assess if a shimming with a dual-transmit system is able to influence breast density assessment on MRI.

In conclusion we found significant differences among MRI sequences for breast density percentage assessment. If the IDEAL WaterOnly sequence is applied as a potential reference, the T1-weighted sequences showed similar breast density percentage values as the reference. Therefore, in clinical practice, the T1-weighted sequence could be a reliable and efficient sequence to measure breast density, and might possibly reflect the true composition of breast tissue.
